# The Role of Vascular Actors in Two Dimensional Dialogue of Human Bone Marrow Stromal Cell and Endothelial Cell for Inducing Self-Assembled Network

**DOI:** 10.1371/journal.pone.0016767

**Published:** 2011-02-03

**Authors:** Haiyan Li, Richard Daculsi, Maritie Grellier, Reine Bareille, Chantal Bourget, Murielle Remy, Joëlle Amedee

**Affiliations:** 1 INSERM U577, Bordeaux and University Victor Segalen Bordeaux 2, Bordeaux, France; 2 IPATIMUP - Instituto de Patologiae Imunologia Molecular da Universidade do Porto, Porto, Portugal; University of Minho, Portugal

## Abstract

Angiogenesis is very important for vascularized tissue engineering. In this study, we found that a two-dimensional co-culture of human bone marrow stromal cell (HBMSC) and human umbical vein endothelial cell (HUVEC) is able to stimulate the migration of co-cultured HUVEC and induce self-assembled network formation. During this process, expression of vascular endothelial growth factor (VEGF_165_) was upregulated in co-cultured HBMSC. Meanwhile, VEGF_165_-receptor2 (KDR) and urokinase-type plasminogen activator (uPA) were upregulated in co-cultured HUVEC. Functional studies show that neutralization of VEGF_165_ blocked the migration and the rearrangement of the cells and downregulated the expression of uPA and its receptor. Blocking of vascular endothelial-cadherin (VE-cad) did not affect the migration of co-cultured HUVEC but suppressed the self-assembled network formation. In conclusion, co-cultures upregulated the expression of VEGF_165_ in co-cultured HBMSC; VEGF_165_ then activated uPA in co-cultured HUVEC, which might be responsible for initiating the migration and the self-assembled network formation with the participation of VE-cad. All of these results indicated that only the direct contact of HBMSC and HUVEC and their respective dialogue are sufficient to stimulate secretion of soluble factors and to activate molecules that are critical for self-assembled network formation which show a great application potential for vascularization in tissue engineering.

## Introduction

In vascularized tissue engineering, formation of blood vessel network is very important: oxygen and nutrient supply will be insufficient due to the lack of blood vessel network [1]; cell loss during the early post-implantational stage causes failure of bone engineering and subsequent bone repair [2]. Vascular endothelial growth factor (VEGF) and basic fibroblast growth factor (bFGF) have been used for stimulating angiogenesis in many reports [3]–[6]. Recently, cell-based approaches have been applied in order to improve tissue vascularization, among which endothelial cells (ECs) have attracted most of the attention [7]–[12].

For studying the angiogenesis process *in vitro* based on endothelial cells, numerous assay models have been applied. Among these models, three-dimensional (3D) assays culturing endothelial cells on a supportive matrix are most common [7]–[9], [13]–[16]. Matrigel, as a matrix derived from murine tumours, has been widely used for *in vitro* tubule formation assay [7], [16]–[17]. However, Donovan et al. demonstrated that the tube formation stimulated by Matrigel are not specific for endothelial cells: several non-endothelial cell types could also be induced to form tube on Matrigel, indicating that the tube formation by endothelial cells on Matrigel may not represent true differentiation of this cell type [17].

Another tube formation *in vitro* assay involving co-culture of endothelial cells with stromal cells with or without matrix has attracted more and more attention [10]–[11], [17]–[21]. Most of these studies co-cultured endothelial cells with fibroblast to promote *in vitro* vasculogenesis [17]– However, Finkenzeller et al. and Fuchs et al. established a co-culture system of human primary osteoblastic cells and human primary endothelial cells to improve neovascularization in bone tissue engineering applications [10]–[11].

Each model has some advantages for studying specific steps involved in the formation of tubular-like network. As our laboratory focused on bone tissue engineering, regenerating bone tissue with not only bone forming cells but also endothelial cells in order to stimulate angiogenesis rapidly and obtain bone reconstruction simultaneously seems attractive. Therefore, we applied a co-culture model where human bone marrow stromal cell (HBMSC) and human umbilical vein endothelial cell (HUVEC) are cultured together in direct contact, trying to mimic the *in vivo* physiological conditions under which angiogenesis is triggered [19]. With this direct contact co-culture model, on the one hand, we found that the co-culture of HBMSC with HUVEC could stimulate the osteoblastic differentiation of HBMSC, which suggested that the co-cultures could stimulate osteogenesis [22]–[27]; on the other hand, we observed that co-cultured HUVEC (co-HUVEC) migrated along co-cultured HBMSC (co-HBMSC) and self-assembled into network structure, which suggested that co-culture of osteoblastic cells with endothelial cells might be able to stimulate angiogenesis in tissue engineering [22]–[23], [25]. However, the cellular and molecular events involved in this co-culture system still remain unclear.

It may be hypothesized that there are complicate and bidirectional cell-cell communications when two different types of cells are co-cultured. For many years, our work is focused on the better understating of this cell cooperation and the role of each cell in the co-culture. There are three major ways for cells to talk each other: the gap junction communications, the adherens and tight junctions and the secretion of diffusible factors that can activate specific receptors on the target cells [28]. Although we have previously studied the roles of connexin-43 and neural-cadherin in the osteoblastic differentiation of HBMSC co-cultured with HUVEC [25], [27], the mode of communication in co-culture system of bone forming cells and endothelial cells that contributes to self-assembled network structure is still unclear.

It has been demonstrated that migration of endothelial cells and formation of tubular-like network structures called capillary cords are critical steps for the angiogenesis and need the participation of adherens molecules [9], [29]–[31]. In endothelial cells, the predominant adherens molecule is vascular endothelial-cadherin (VE-cad), which is cell-specific and strictly located at the junctions of endothelial cells [32]. The role of VE-cad in determining the endothelial cell contact integrity, controlling the cellular junction and tubular-like network formation has been extensively demonstrated through functional studies in monocultures of endothelial cells [5], [33]–[35]. Bach et al. reported that inhibition of VE-cad not only blocked the generation of capillary tubes but also disrupted the preformed tubes [7]. Corada et al. found that the paracellular permeability was increased and angiogenesis *in vitro* was blocked when endothelial cells were cultured with VE-cad neutralizing antibodies [33].

In addition, our previous studies demonstrated that expression of VEGF_165_ was significantly upregulated in co-HBMSC [22]. VEGF has been reported in many studies on mono-cultured endothelial cells that it could promote angiogenesis through stimulating migration of mono-cultured endothelial cells, inducing the phosphorylation and redistribution of adhesion junction molecules, or increasing the expression of proteolytic enzyme [36]–[40]. It is also well known that cells have to express proteolytic enzymes allowing cleavage of matrix proteins for favouring cell migration [41]–[42]. Although both of serine proteases of plasminogen/plasmin system and matrix metalloproteinases are important as proteolytic enzymes, the key enzyme of the proteolytic machinery is uPA, which is produced *via* a receptor-bound conversion of pro-uPA to active uPA [41], [43]. Many studies on mono-cultured endothelial cells have reported that VEGF_165_ plays an important role in this proteolytic process. Prager et al. demonstrated that VEGF_165_ initiated proteolysis by activation of pro-uPA *via* the VEGF-receptor 2 (KDR) and uPAR redistributed to focal adhesions at the leading edge of endothelial cells in response to VEGF_165_ [42].

However, although these molecules have been demonstrated since many years that they are involved in tubular-like network formation in mono-cultured endothelial cells in 2D cultures or in 3D systems, as far as we know, only role of VEGF on tube formation in co-culture of endothelial cells and fibroblasts has been reported [21], [44]. The roles of uPA, uPAR and VE-cad in tubular-like network formation in a co-culture system are still unclear. Based on the importance of VEGF, uPA and uPAR in mono-cultured endothelial angiogenesis [36]–[42] and our previous findings in co-culture system about the functions of VEGF and cadherin [22], [25], we hypothesize that these molecules should play an important role in the self-assembled network formation in a co-culture of human stromal cells and endothelial cells.

Therefore, for the first time, we investigated the function links of VE-cad, KDR, VEGF_165_, uPA and uPAR in the communication between HBMSC and HUVEC which were co-cultured in 2D and in direct contact, trying to explain the contribution of each type of cells in the self-assembled network formation and the roles of key vascular molecules in the self-assembled network formation. We found that, in the co-culture system, co-HBMSC was responsible for producing VEGF_165_ to activate its receptor-KDR in co-cultured HUVEC. With the expression of VEGF_165_ and KDR, the expression of proteolytic enzymes in co-HUVEC was stimulated and the proteolytic machinery was initiated. Therefore, the co-HUVEC could migrate and self-assembled into network structure. VE-cad was found to be necessary for the self-assembled network formation although it did not affect the migration of co-HUVEC.

## Results

### Expression of VE-cad during self-assembled network formation in co-culture

**Figure 1:**
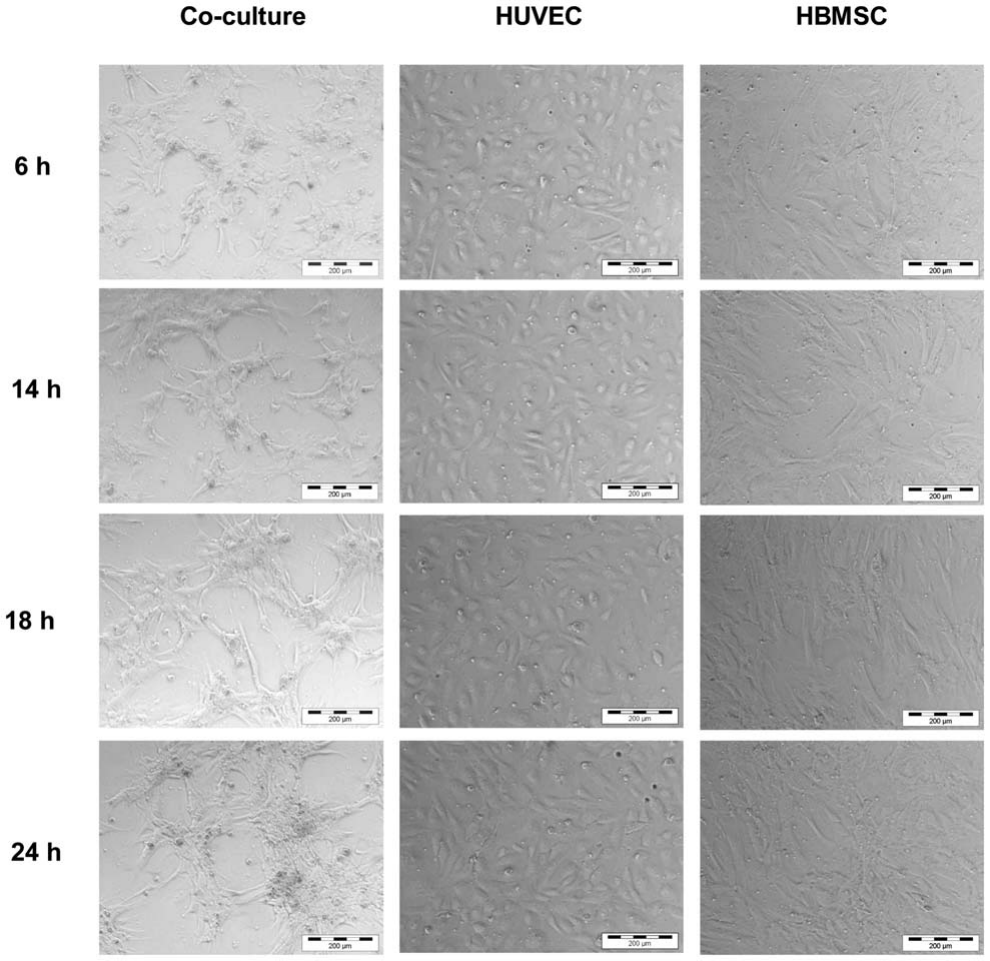
Light microscopy observation on HBMSC, HUVEC, co-HBMSC and co-HUVEC. Self-assembled network formed in co-cultures of HBMSC and HUVEC after 6 hours, 14 hours, 18 hours, and 24 hours (Arrows). No self-assembled network formed in mono-cultured HBMSC and HUVEC. Scare bars represent 200 µm.

In our previous studies, it has been demonstrated that self-assembled network could be observed only in co-cultures of HBMSC and HUVEC after 24 hours [22]–[23], [25]. Here, the time course process of the self-assembled network formation in the co-cultures has been investigated and the results are shown in Fig. 1. It can be seen that cells self-assembled during 6 to 24 hours and formed network structure at 24 hours, as shown in Fig. 1 (left column). On the contrary, no self-assembled network formation could be observed in mono-cultured HBMSC and HUVEC.

**Figure 2:**
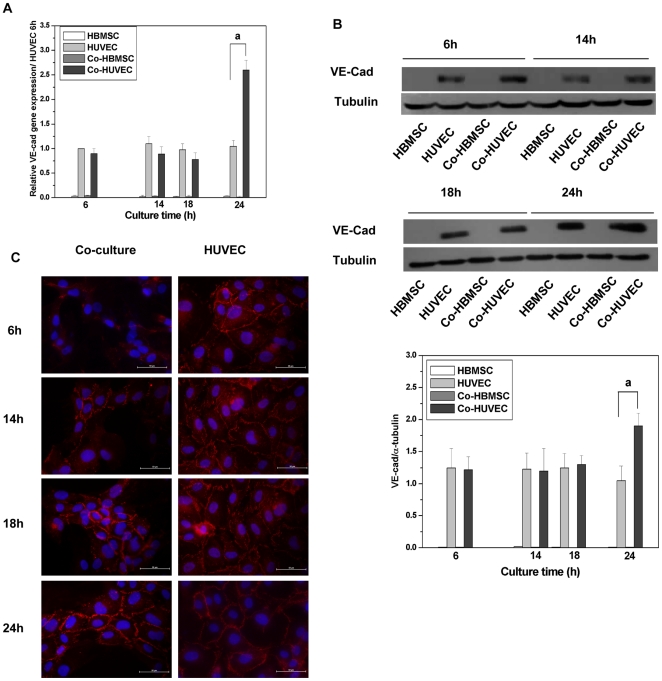
Expression of VE-cad in HBMSC, HUVEC, co-HBMSC and co-HUVEC after being cultured for different time. (A) The VE-cad expression was detected by Q-PCR for mRNA level. Data of gene expression was quantified relative to VE-cad gene expressions of HUVEC after 6 h of culture. During 6 hours–18 hours, the gene expression of VE-cad kept constant in HUVEC and co-HUVEC and there is no significant difference of VE-cad gene expression in HUVEC as compared that in co-HUVEC. However, a significant increase of VE-cad gene expression in co-HUVEC was detected at 24 hours while the gene expression of VE-cad in HUVEC still maintained constant. (B) The VE-cad expression was detected by western blot for obtaining protein level. For western blot assay, α-tubulin was analyzed as loading control. The analysis was carried out based on three independent experiments and the gel photos were taken as a representative (not show the other two experiment gel photos). There is no VE-cad detected in co-HBMSC, which indicated the separation of co-cultured cells was completed. **a** indicated the difference p≤0.05. VE-cad protein expression confirmed the results of gene expression, which showed that there is no significant difference of VE-cad expression between HUVEC and co-HUVEC during 6 hours–18 hours but the co-HUVEC expressed much higher VE-cad than HUVEC at 24 hours. (C) Immunofluorensence staining of VE-cad in co-HUVEC and HUVEC in red. Nuclear was stained in blue with DAPI. Scare bars represent 50 µm. VE-cad started to show at the junction of co-HUVEC from 14 hours and located at the junction of HUVEC all the time.

To further understand the role of VE-cad on the self-assembled network formation, the expression of VE-cad in HBMSC, HUVEC, co-HBMSC and co-HUVEC at different time points was detected after the co-cultured cells were separated (Fig. 2). We can see that there is no significant difference between the expression of VE-cad in HUVEC and co-HUVEC during the development of self-assembled network (6 hours, 14 hours, and 18 hours), which were detected by Q-PCR and western blot (Fig. 2A, B). The expression of VE-cad kept constant in HUVEC and co-HUVEC during 6 hours-18 hours but significantly increased in co-HUVEC when the self-assembled network formed at 24 hours. Therefore, co-HUVEC expressed much more VE-cad than HUVEC at 24 hours. No VE-cad could be detected in HBMSC or co-HBMSC, which indicated that the separation of co-cultured cells was successfully. Interestingly, immunofluorescence staining of VE-cad (in red) on co-HUVEC showed that very few VE-cad was detected at the junctions of cells after 6 hours and 14 hours of co-culture. At 18 hours, VE-cad appeared at junctions of the co-HUVEC. When time increased, more and more VE-cad (stained in red) appeared at the junctions of co-HUVECs (Fig. 2C left column). The mono-cultured HUVEC kept confluence and VE-cad appeared at cell junctions all the time (Fig. 2C right column), whereas there is no VE-cad expression in HBMSC (data not shown).

### Expressions of VEGF_165_, KDR, uPA and uPAR were regulated in co-culture

**Figure 3:**
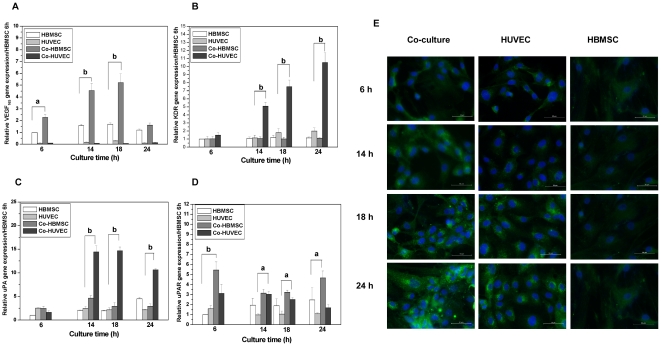
Expression of VEGF_165_, KDR, uPA and uPAR in HBMSC, HUVEC, co-HBMSC and co-HUVEC after being cultured for different time. (A) Gene expression VEGF_165_ in HBMSC, HUVEC, co-HBMSC and co-HUVEC after being cultured for different time. Data of gene expression was quantified relative to the same gene expression in HBMSC after 6 hours of culture (same in the following gene expression analysis). a and b indicated the difference p≤0.05 or p≤0.01, respectively. Interestingly, we found that VEGF_165_ was only expressed by HBMSC and co-HBMSC and VEGF_165_ expression in co-HBMSC is much higher than that in HBMSC during 6 hours–18 hours. However, this upregulation in co-HBMSC diminished at 24 hours. (B) Gene expression of KDR in HBMSC, HUVEC, co-HBMSC and co-HUVEC after being cultured for different time. KDR was mainly expressed by endothelial cells and it was much higher in co-HUVEC than in HUVEC from 14–24 hours. At 6 hours, no significant difference has been observed. (C) Gene expression of uPA in HBMSC, HUVEC, co-HBMSC and co-HUVEC after being cultured for different time. uPA has a similar expression style as to the expression of KDR. (D) Gene expression of uPAR in HBMSC, HUVEC, co-HBMSC and co-HUVEC after being cultured for different time. uPAR expressed by all cells and it is higher expressed by co-cultured cells as compared to mono-cultured cell. (E) Immunofluorescence staining of KDR in cells in green. Nuclear was stained in blue with DAPI. Scare bars represent 50 µm. It can be clearly seen that the expression of KDR is much higher in co-cultured cells than in mono-cultured HUVEC. No much KDR could be detected in HBMSC by immunofluorescence.

We further investigated the expression of VEGF_165_, KDR, uPA and uPAR in HBMSC, HUVEC, co-HBMSC and co-HUVEC during the self-assembled network formation. VEGF_165_ was not expressed in HUVEC or co-HUVEC. However, we found that the VEGF_165_ was expressed in HBMSC and the expression of VEGF_165_ was significantly upregulated in co-HBMSC as compared with that in HBMSC. Interestingly, the expression of VEGF_165_ in co-HBMSC reached peaks at 14 hours and 18 hours followed by a sharp decrease at 24 hours (Fig. 3A). Meanwhile, the gene expression of KDR was significantly upregulated in co-HUVEC as shown in Fig. 3B and confirmed by immunofluorescence staining of KDR (in green) (Fig. 3E). Fig. 3C shows that uPA was mainly synthesized in co-HUVEC and the upregulation of uPA expression in co-HUVEC began at 14 hours and maintained at 18 hours followed by a slight decrease at 24 hours. At 14 hours and 18 hours, the expression of uPA in co-HUVEC was about threefold higher than that in HUVEC. We also found that uPAR was expressed by all cells and it was higher expressed in both co-HBMSC and co-HUVEC than in mono-cultured cells (Fig. 3D).

### Involvement of VE-cad, VEGF_165_, and uPAR in cell migration and self-assembled network formation

**Figure 4:**
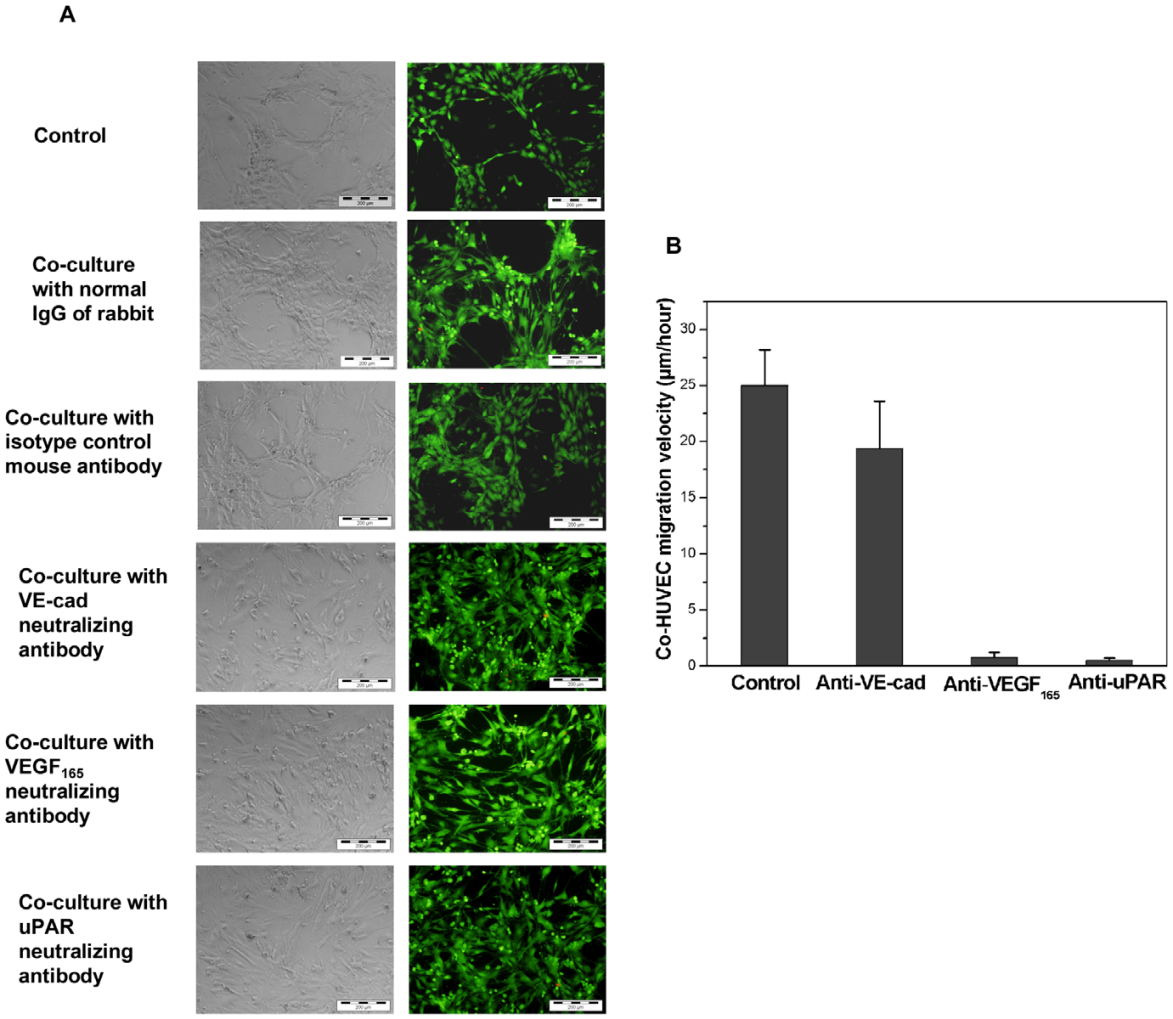
Functional studies of VE-cad, VEGF_165_ and uPAR. (A) HBMSC and HUVEC were co-cultured with neutralizing antibodies against VE-cad, VEGF_165_ and uPAR for 24 hours. We found that the neutralization of VE-cad, VEGF_165_ and uPAR suppressed the formation of self-assembled network in co-culture system without impairing the viability of cells. Addition of isotype control mouse antibody or normal IgG rabbit antibody had no effect on self-assembled network formation. Scare bars represent 200 µm. (B) The migration velocity of co-HUVEC were analyzed from the images taken by time-lapse videomicroscopy observing co-cultures with or without neutralizing antibody. Interestingly, co-HUVEC in co-culture incubated with VE-cad neutralizing antibody was still be able to migrate. The migration speed is very close to the co-HUVEC in co-culture without any neutralizing antibody. However, the neutralization of VEGF_165_ and uPAR totally blocked the migration of co-HUVEC in co-culture system.

To further understand the exact role of VE-cad, VEGF_165_ and uPAR in the self-assembled network formation, functional studies have been performed here using neutralizing antibodies which have been demonstrated as efficient probes for studying these molecules [5], [33]–[35], [45]–[48]. Migration of co-HUVEC and the self-assembled network formation in the co-cultures with or without these neutralizing antibodies were detected by time-lapse videomicroscopy. It can be seen from Fig. 4A that neutralization of VE-cad, VEGF_165_ and uPAR successfully suppressed the self-assembled network formation (left column) without impairing the cell viability (right column). Addition of isotype control mouse antibodies or IgG control rabbit antibody had no effect on the formation of self-assembled network. Interestingly, neutralization of VE-cad did not stop the migration of co-HUVEC while the neutralization of VEGF_165_ and uPAR totally blocked the migration of co-HUVEC in co-culture system (Fig. 4B).

**Figure 5:**
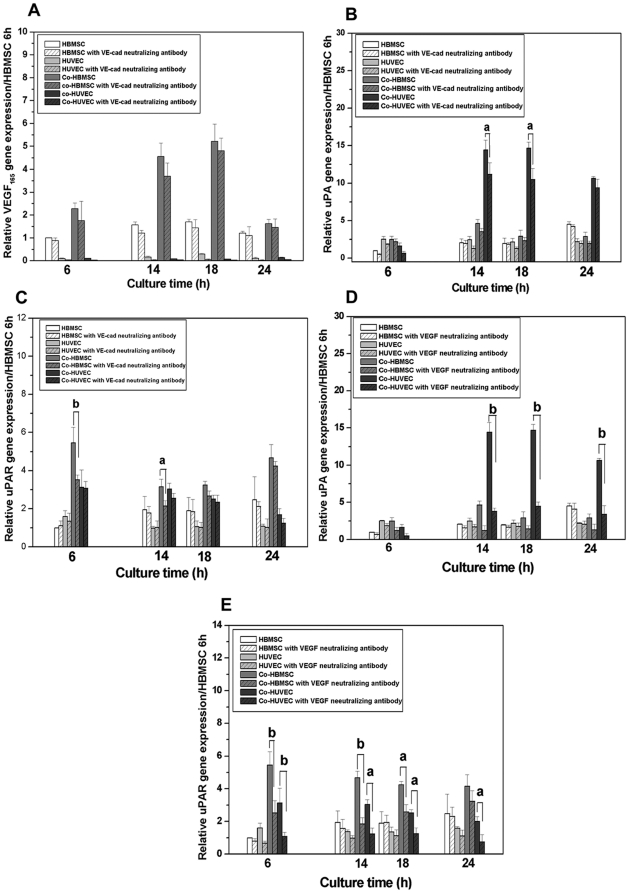
Gene expressions of VEGF_165_, uPA and uPAR in functional studies. (A) Gene expressions of VEGF_165_ in HBMSC, HUVEC, Co-HBMSC and co-HUVEC cultured with (bars with shadow) or without VE-cad neutralizing antibody. Neutralization of VE-cad has no strong effects on expression of VEGF_165_. (B) Gene expressions of uPA in HBMSC, HUVEC, Co-HBMSC and co-HUVEC cultured with (bars with shadow) or without VE-cad neutralizing antibody. Neutralization of VE-cad significantly downregulated the expression of uPA in co-HUVEC at 14 hours and 16 hours and this effect decreased at 24 hours. However, the expression of uPA in co-HUVEC still maintained a high level. (C) Gene expressions of uPAR in HBMSC, HUVEC, Co-HBMSC and co-HUVEC cultured with (bars with shadow) or without VE-cad neutralizing antibody. uPAR was transiently affected by neutralization of VE-cad. Its expression statistically decreased in co-HBMSC at 6 hours and 14 hours. (D) Gene expression of uPA in HBMSC, HUVEC, Co-HBMSC and co-HUVEC with (bars with shadow) or without VEGF_165_ neutralizing antibody. Neutralization of VEGF_165_ strongly suppressed the expression of uPA in co-HUVEC. (E) Gene expression of uPAR in HBMSC, HUVEC, Co-HBMSC and co-HUVEC with (bars with shadow) or without VEGF_165_ neutralizing antibody. Gene expression of uPAR in co-cultured cells was downregulated all the time after the addition of VEGF_165_ neutralizing antibody. **a** and **b** indicated the difference p≤0.05 or p≤0.01, respectively.

To further understand the role of VE-cad in the migration of co-HUVEC and self-assembled network formation in the co-culture, we detected the expression of VEGF_165_, uPA and uPAR in the mono-cultured or co-cultured cells incubated with VE-cad neutralizing antibody in order to see if VE-cad has strong effects on these molecules' expression. It can be seen from Fig. 5A that neutralization of VE-cad has not significantly affected the expression of VEGF_165_ in all cells but downregulated the expression of uPA in co-HUVEC and uPAR in co-HBMSC transitionally (Fig. 5B, C). However, the expression of uPA still maintained a relative high level even after the downregulation (Fig. 5B). Different from the mild effects of VE-cad on the expression of uPA and uPAR in all cells, neutralization of VEGF_165_ significantly suppressed the expression of uPA and uPAR in co-HBMSC and co-HUVEC and the suppression started from 6 hours, affecting the cells all the time except in co-HBMSC at 24 hours (Fig. 5D, E).

## Discussion

Recently, co-cultures of endothelial cells with other cell types have attracted more and more attention for studying angiogenesis [10]–[11], [18]–[19], [21], [44], [49]–[54]. Most investigators chose fibroblast to be co-cultured with endothelial to promote vascularisation with or without stimulation by VEGF [18], [21], [49]–[54]. Focusing on bone tissue engineering, our laboratory has been working on the co-cultures of HBMSC and HUVEC for about ten years to investigate the effects of HBMSC-HUVEC interactions on osteoblastic differentiation of HBMSC and angiogenesis of endothelial cells [22]–[27]. In this study, we further study the key molecules actors involved in self-assembled network formation in co-culture of HBMSC and HUVEC.

Although the molecules involved in angiogenesis and tube formation are extensively studied in mono-cultured endothelial cells or associated with a 3D matrix (Matrigel, for example), nothing is known in a co-culture system where endothelial cells form straight contacts with human stromal cells. There are at least 3 major mechanisms that are involved in endothelial cell migration: (1) chemotaxis, the migration being directed by a gradient of soluble chemoattractants; (2) haptotaxis, the migration being directed by a gradient of immobilized ligands; (3) mechanotaxis, the migration generated by mechanical forces [31]. In addition to the above 3 major mechanisms that involve in endothelial cell angiogenesis, the function of cadherin in migration of cells and capillary tube formation has attracted more and more attention in angiogenesis research [5], [33]–[35]. However, all of these studies are also based on mono-cultured endothelial cells or 3D system [7], [55]–[57].

Here, we studied the role of VE-cad in the self-assembled network formation in 2D co-culture of HBMSC and HUVEC. Although VE-cad expression has not changed during the development of self-assembled network (6 hours to 24 hours) (Fig. 2) and neutralization of VE-cad has slight effects on the expression of VEGF, uPA and uPAR (Fig. 5A–C), the dynamic localization of VE-cad was noticeable: it disappeared from the junctions of co-HUVEC at 6 hours and 14 hours and appeared again at the junctions of co-HUVEC at 18 hours and 24 hours (Fig. 2).

Loss of VE-cad in cell-cell junctional regions has been reported in many angiogenesis studies [6], [32]. Wright et al. has reported that complex of VE-cad and beta (β)-catenin were lost from adherens junctions until recruited back to cell-cell contacts during the latter stages of angiogenesis induced by type I collagen gel [6]. The dissociation of complex of VE-cad and β-catenin from junctional regions has been observed during junction remodelling in endothelial cells subjected to fluid shear stress [32]. Interestingly, the disappearance of VE-cad from junctional regions was not due to a downregulation of expression or a degradation of VE-cad. The reasons that induced the temporary loss of VE-cad from the junctions have not been fully elucidated but the phosphorylation of VE-cad induced by VEGF_165_ might reduce the strength of its association to the actin cytoskeleton, allowing VE-cad and β-catenin to remain sequestered by the membrane and the rapid return of this complex to adherens junctions when required [31]. In our opinion, this kind of loss-turnover redistribution of VE-cad might facilitate the migration of HUVEC and the self-assembled network formation: the disappearance of VE-cad could disorganize the adherens junctions between HUVEC, which favoured the cells to migrate; the turnover of VE-cad could tighten the adherens junctions between HUVEC, which ensured the self-assembled network to be formed.

Because the use of SiRNA introduces additional difficulties (mainly the low transfection efficiency in human primary cells or human stromal cells) while the efficiency of neutralizing antibodies have been proved in previous studies [5], [33]–[35], [45]–[48], we have selected several neutralizing antibodies against VE-cad, VEGF and uPAR for functional studies. Neutralization of VE-cad has light effects on the expression of uPA and uPAR and the effects gradually diminished during the self-assembled network formation, which might explain the migration of co-HUVEC even after the treatment of VE-cad neutralizing antibody. However, the migration was random, which was not like the migration of the co-HUVEC observed in the co-cultures without VE-cad neutralizing antibody. In addition, we found that there was no self-assembled network formation when the co-culture was treated with VE-cad neutralizing antibody even there was still migration of co-HUVEC. As lacking of VE-cad could disorganize the adherens junctions between co-HUVEC, the migrating cells might be not able to adhere with each other and self-assembled into network structure.

As we mentioned above, chemotaxis is one of the major mechanisms involved in endothelial cell angiogenesis. Typically, chemotaxis of endothelial cells is driven by growth factors such as VEGF and bFGF [58]. VEGF is an angiogenic growth factor possessing many special properties to induce endothelial cells to proliferate, to migrate, to assemble into tubes, to survive and to increase their permeability: VEGF is normally produced by cells surrounding endothelial cells in close proximity; VEGF affects endothelial cells *via* interactions with cellular receptors KDR and VEGF receptor-1 [40], [59].

In this study, the gene expression showed that VEGF_165_ was mainly produced by HBMSC and it was upregulated in co-HBMSC. However, in our previous and current studies, VEGF protein was not detected in medium of co-culture while it was quantified in HBMSC co-cultured with fibroblast cells [22]. We assumed that the VEGF_165_ produced by co-HBMSC might be consumed by co-HUVEC. Therefore, VEGF_165_ could act as a chemotaxis and induced the co-HUVEC migration and self-assembled network formation in a paracrine manner in our co-culture system. In addition, Hurley et al. recently reported a complex temporal regulation of capillary morphogenesis by fibroblasts in a co-culture of fibroblast and endothelial cells [44]. They demonstrated that fibroblasts enhanced early capillary network formation by assisting the endothelial cell migration and increasing the VEGF and angiopoietin-1 expression but the effects were temporary. Our study also found that the VEGF_165_ expression largely decreased in co-HBMSC after 24 hour culture as compared to those at 14 and 18 hours, indicating that the VEGF effects on cell migration and self-assembled network formation might be transiently.

Roles of uPA and uPAR in migration and self-assembled network formation have also been extensively studied in mono-cultures and have been commonly considered as proteolytic enzymes for degradation of matrix to initiate cell migration [41]–[42], [60]. In the present study, expression of uPA was significantly enhanced in co-HUVEC and expression of uPAR was significantly upregulated in all co-cultured cells, which might be a reason for the migration of co-HUVEC and self-assembled network formation in the co-culture. In addition, the enhanced expression of uPA in co-HUVEC might be in response to the upregulation of VEGF_165_ in co-HBMSC, which further confirmed the chemotaxis effects of VEGF_165_ on the migration of HUVEC and the self-assembled network formation.

Functional studies showed that neutralization of VEGF_165_ and uPAR totally blocked the endothelial cell migration and self-assembled network formation in the co-culture. Neutralization of VEGF_165_ resulted in the downregulation of uPA and uPAR gene expression, indicating that the overexpression of proteolytic enzyme in co-cultured cells was induced by VEGF_165_ and suggesting that the migration of co-HUVEC and self-assembled network formation in co-cultures of HBMSC and HUVEC needed the participation of VEGF_165_ and uPA/uPAR.

In conclusion, in this study, we used a direct contact co-culture model of HBMSC and HUVEC to study the key molecules involved in the self-assembled network formation. Our findings demonstrated that co-culture of HBMSC and HUVEC could stimulate the migration of co-cultured HUVEC and induce the self-assembled network formation through their contact and specific dialogue. Here, the only direct contact of HBMSC and HUVEC was sufficient to stimulate the secretion of key soluble factors that are critical for self-assembled network structure: VEGF_165_ was upregulated in co-HBMSC, which in turn activated uPA through uPAR in co-HUVEC, probably triggering the proteolytic machinery and initiating the migration of co-HUVEC and self-assembled network formation. As adherens molecular, VE-cad is necessary for the self-assembled network formation. These new data in the field of cell to cell communication between stromal cells and endothelial cells may have implications in study of angiogenesis in bone tissue engineering.

## Materials and Methods

### Cell culture

Human bone marrow stromal cell (HBMSC) [25]–[26] and human umbilical vein endothelial cell (HUVEC) [25]–[26] were obtained and cultured according to methods described previously [22], [25]–[26], [61]–[62]. Cells were either mono-cultured or co-cultured for 24 h and samples were taken at different time points for measurements. For immunofluorescence detection, cells were cultured on glass coverslips in 24-well plates at the densities of 20,000 HBMSCs/cm^2^ and 40,000 HUVECs/cm^2^. Self-assembled network formation was monitored by phase contrast microscopy (Zeiss Axiovert 25, Seli, France).

### Cell separation after co-culturing using magnetic beads

As VE-cad is particularly sensitive to trypsin in absence of Ca^2+^, mono-cultured HUVEC and co-cultured HUVEC were detached in a way described before with minor modifications to maximally preserve VE-cad at the cell surface and to avoid its degradation [5]. HUVEC were separated from HBMSC by applying magnetic beads coupled with an antibody against CD31, a specific protein of ECs according to the methods established by Guillotin et al [23].

### Quantitative real time polymerase chain reaction (Q-PCR)

Total RNA was prepared from cells using Total RNA Isolation kit (NucleoSpin ® RNA 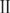, MACHEREY-NAGEL) according to the manufacture's guidelines. Complementary DNA (cDNA) was synthesized according to the protocols established by Grellier et al. [22]. Primers of VE-cad, VEGF_165_, KDR, uPA and uPAR (all from Eurogentec) were used as the final concentration of 250 nM; their sequences are reported in Table 1. Data were analyzed with the iCycler IQTM software and compared by the ΔΔCt method and each Q-PCR was performed in triplicate for PCR yield validation. Data was normalized to P_0_ mRNA expression for each condition and was quantified relative to VEGF_165_, KDR, uPA and uPAR gene expressions of HBMSC after 6 h of culture or relative to VE-cad gene expression of HUVEC after being cultured for 6 h, which were standardized to 1.

**Table 1:** Primer sequences used in Q-PCR.

Transcript	GenBank	Primer sequences	TM (°C)
VE-cad	NM 001795	forward 5′ GGC TCA GAC ATC CAC ATA ACC 3′reverse 3′ CTT ACC AGG GCG TTC AGG GAC 3′	63
VEGF_165_	AB021221	forward 5′ TAT GCG GAT CAA ACC TCA CCA 3′reverse 5′ CAC AGG GAT TTT TCT TGT CTT GCT 3′	58
uPA	NM 001145031.1	forward 5′ CAC GCA AGG GGA GAT GAA 3′	60
		reverse 5′ ACA GCA TTT TGG TGG TGA CTT 3′	
uPAR	NM 001005376.1	forward 5′ GCCCAATCCTGGAGCTTGA 3′	60
		reverse 5′ TCCCCTTGCAGCTGTAACACT 3′	
P_0_	BC015690	forward 5′ ATG CCC AGG GAA GAC AGG GC 3′reverse 5′ CCA TCA GCA CCA CAG CCT TC 3′	65

### Immunofluorescence

KDR (VEGF-R2) and VE-cad were detected by immunofluorescence staining according to the procedures described in our previous study [25] using a mouse anti-VEGFR2 primary antibody and a mouse anti-VE-cadherin primary antibody, respectively (BD Bioscience Pharmingen). Alexa 488 goat anti-mouse IgG or Alexa 568 goat anti-mouse IgG secondary antibody (Invitrogen) was used for revealing KDR and VE-cad in green and red, respectively. Nuclei were revealed with 1 µg/ml 4′6-diamidion-2-phenylindole (DAPI, FluoProbes) for 10 min at room temperature. Cells were then observed with a fluorescence microscope (Nikon Eclipse 80i, Japan) and images were taken by a digital camera (Nikon Dxm 1200C, Japan).

### Western blot

Protein extraction from cells was performed according to previous description [23], [25]. Quantification of the protein was performed using BCA (bicinchoninic acid) protein assay kit (Pierce, Perbio Science, Bezons, France).

Western blot analysis was carried out as previously described [25]. Membranes were incubated with a primary rabbit anti-VE-cad antibody (Cell Signalling Technology) diluted at 1/1000 in blocking buffer (TBS-T: 20 mM Tris HCl, pH 7.4, 150 mM NaCl, 0.05% (v/v) Tween 20) containing 5% (w/v) non fat milk). Loading control was performed by incubating membrane with a mouse monoclonal antibody against α-tubulin (Sigma) diluted at 1/30000 in blocking buffer. Immunoreactive bands were visualized using horseradish-peroxidase-conjugated secondary antibodies (HRP, goat anti-rabbit, Jackson ImmunoResearch Laboratories, Inc.), or HRP goat anti-mouse for α-tubulin (Chemicon, Euromedex, France) diluted at1/15000 in blocking buffer. Membrane was immersed in enhanced chemiluminescence detect reagent (ECL-plus, Amersham, BioSciences, France) and exposed to KODAK photographic film. The intensities of the bands were quantified by a Bio Imaging System (Gene Genius, Syngene) with GeneTools software.

### Time-lapse videomicroscopy

To find out the critical molecules for the migration of co-HUVEC and the formation of self-assembled network, we blocked VE-cad, VEGF_165_ and uPAR with neutralizing antibody and monitored the migration of co-HUVEC and the formation of self-assembled network in co-culture system by time-lapse videomicroscopy. Time lapse videomicroscopy was performed according to the method described by Grellier et al. with slight modifications [22]. Briefly, HUVEC was labelled with Dil-Ac-LDL (1,1′-dioctadecyl-3,3,3′,3′-tetramethylindocarbocyanine perchlorate-Acetylated-Low Density Lipoprotein, Harbor Bioproducts, USA) by incubating in culture medium containing 0.2 µg/ml of Dil-Ac-LDL overnight. Then, HUVEC stained with Dil-Ac-LDL and HBMSC were seeded in a special culture chamber (Glass base dish, IWAKI, Japan) and incubated at 37°C in a 5% CO_2_ humid atmosphere with IMDM-10% (v/v) FBS for 1 hour before the chamber was settled on a thermostable plate sitting on the microscope (Leica, TCS SP5), which allows for a stable temperature of 37°C in the chamber with 5% CO_2_ humid atmosphere. The microscope was programmed to take an image every 10 minutes for 12 hours in transmission and fluorescence. The obtained serial images could be reconstructed into movies by the LAS-AF (Leica Advanced Suite-Advanced Fluorescence) software. The migration speed of co-HUVEC was obtained through calculating and averaging the migration speed of every single cell by using Image J software (NIH). Three independent experiments were performed for each neutralizing antibody.

### Functional studies

For neutralization experiments, HBMSC and HUVEC were separately pre-treated with proper antibodies overnight before they were cultured on glass coverslips or in 25 cm^2^ flasks. As controls, non-pre-treated cells were cultured without the neutralizing antibody. Mouse monoclonal antibody against human VE-cad (Hycult Biotechnology) was used at 15 µg/ml, rabbit polyclonal antibody against human VEGF_165_ (Millpore) was used at 10 µg/ml and rabbit polyclonal antibody against human uPAR (American Diagostica Inc.) was used at 5 µg/ml. Isotype mouse antibody or normal IgG rabbit antibody have been used as control antibodies. Live-dead assay was applied to the cells cultured with or without neutralizing antibody to confirm their viability using ethidium homodimer and calcein-AM regents.

### Statistical analysis

Data were expressed as means ± standard deviation (SD) for n = 3 (three independent experiments) and were analyzed using standard analysis of Student's *t*-test. Differences were considered significant when *p*≤0.05 (**a**) or *p*≤0.01 (**b**).
